# Transcriptomic insights into the early host-pathogen interaction of cat intestine with *Toxoplasma gondii*

**DOI:** 10.1186/s13071-018-3179-8

**Published:** 2018-11-14

**Authors:** Meng Wang, Fu-Kai Zhang, Hany M. Elsheikha, Nian-Zhang Zhang, Jun-Jun He, Jian-Xun Luo, Xing-Quan Zhu

**Affiliations:** 1 State Key Laboratory of Veterinary Etiological Biology, Key Laboratory of Veterinary Parasitology of Gansu Province, Lanzhou Veterinary Research Institute, Chinese Academy of Agricultural Sciences, Lanzhou, Gansu Province 730046 People’s Republic of China; 2 Faculty of Medicine and Health Sciences, School of Veterinary Medicine and Science, University of Nottingham, Sutton Bonington Campus, Loughborough, LE12 5RD UK; 3 Jiangsu Co-innovation Center for the Prevention and Control of Important Animal Infectious Diseases and Zoonoses, Yangzhou University College of Veterinary Medicine, Yangzhou, Jiangsu Province 225009 People’s Republic of China

**Keywords:** *Toxoplasma gondii*, Cat, RNA-Seq, Gene expression, Immune response, Xenobiotic metabolism

## Abstract

**Background:**

Although sexual reproduction of the parasite *Toxoplasma gondii* exclusively occurs in the cat intestine, knowledge about the alteration of gene expression in the intestine of cats infected with *T. gondii* is still limited. Here, we investigated the temporal transcriptional changes that occur in the cat intestine during *T. gondii* infection.

**Methods:**

Cats were infected with 100 *T. gondii* cysts and their intestines were collected at 6, 12, 18, 24, 72 and 96 hours post-infection (hpi). RNA sequencing (RNA-Seq) Illumina technology was used to gain insight into the spectrum of genes that are differentially expressed due to infection. Quantitative RT-PCR (qRT-PCR) was also used to validate the level of expression of a set of differentially expressed genes (DEGs) obtained by sequencing.

**Results:**

Our transcriptome analysis revealed 2363 DEGs that were clustered into six unique patterns of gene expression across all the time points after infection. Our analysis revealed 56, 184, 404, 508, 400 and 811 DEGs in infected intestines compared to uninfected controls at 6, 12, 18, 24, 72 and 96 hpi, respectively. RNA-Seq results were confirmed by qRT-PCR. DEGs were mainly enriched in catalytic activity and metabolic process based on gene ontology enrichment analysis. Kyoto Encyclopedia of Genes and Genomes pathway analysis showed that transcriptional changes in the intestine of infected cats evolve over the course of infection, and the largest difference in the enriched pathways was observed at 96 hpi. The anti-*T. gondii* defense response of the feline host was mediated by Major Histocompatibility Complex class I, proteasomes, heat-shock proteins and fatty acid binding proteins.

**Conclusions:**

This study revealed novel host factors, which may be critical for the successful establishment of an intracellular niche during *T. gondii* infection in the definitive feline host.

**Electronic supplementary material:**

The online version of this article (10.1186/s13071-018-3179-8) contains supplementary material, which is available to authorized users.

## Background

Toxoplasmosis, caused by the intracellular parasite *Toxoplasma gondii*, is a disease of global importance. *Toxoplasma gondii* has been reported in nearly all warm-blooded animals [1]. However, its only definitive host is the cat, including members of the family Felidae, where the sexual cycle occurs in the enterocytes of the small intestine. Cat infection occurs *via* the ingestion of bradyzoite-containing tissue cysts, and following excystation of bradyzoites in the gut, the parasites infect the epithelium and progress from the asexual merozoite stage into sexual stages called gametes (micro- and macrogametocytes). Male and female gametes fuse to form microscopic zygotes/oocysts, which are excreted in cat feces into the environment where they mature into sporulated oocysts [2]. Infected cats can produce millions of oocysts through a single infection [3]. These environmentally resistant oocysts can remain infectious for long periods of time in the environment [4, 5]. When ingested by intermediate hosts, infectious sporozoites are liberated from the oocysts and differentiate into tachyzoites in order to complete the life-cycle.

Humans (an intermediate host) can be infected not only *via* eating meat containing parasite cysts, but also *via* accidental ingestion of water contaminated with oocysts [6], ingestion of oocysts in contaminated food sources [7], or *via* gardening or cleaning cat litter. Although *T. gondii* does not seem to cause major illness in healthy people [8], patients with an immunocompromised status, such as with AIDS or cancer may develop fatal encephalitis, mainly because of the reactivated tissue cysts [9]. *Toxoplasma gondii* can also cause serious health complications in an unborn child if the primary infection occurred during pregnancy. Other intermediate hosts, such as herbivorous and omnivorous animals, are infected by eating oocysts in contaminated food sources or water supplies [1].

Despite the crucial epidemiological role played by *T. gondii* oocysts in the dissemination of infection to humans and animals [10], studies investigating the gene regulatory pathways that are involved in the early stages of feline intestinal infection and the formation of oocysts are still limited. The intestinal epithelium can function as a physical barrier to protect the host against microbial infection [11, 12], and also participates in host innate immunity through the production of cytokines, chemokines and antimicrobial peptides [13–15]. Thus, enhanced understanding of the host-pathogen interactions that permit *T. gondii* to develop into sexual stages in the cat intestine would help advance the development of improved preventative and therapeutic approaches to thwart the infection at the main source in the definitive host.

The lack of knowledge about the transcriptional changes that occur in cat intestine during early stage of *T. gondii* infection has prompted us to map the temporal changes that occur in the cat intestinal transcriptome during the first 96 hours after infection. In this study, we used RNA sequencing to illustrate the transcriptional changes that occur in the intestine of cats during early stages of *T. gondii* infection. Our findings provide new insight into defense strategies in the feline intestine and uncover host genes modulated during infection, including genes required for host defense and genes required for the growth of the parasite.

## Methods

### Animals

Female (2- to 3-month-old) domestic cats (*Felis catus*) of the Chinese Li Hua breed were purchased from a local breeder and housed in a controlled environment. Prior to the experiment, all cats were confirmed to be negative from *T. gondii* using the modified agglutination test (MAT) and free of major viral infections (e.g. feline immunodeficiency virus, feline parvovirus, feline calicivirus, feline coronavirus and feline leukemia virus) based on serological examination. Cats received commercial cat diets (Royal Canine Inc., St. Charles, USA) and water *ad libitum* during the two weeks prior to experimentation in order to minimize any potential dietary impact on the study results. After challenging with *T. gondii*, each cat was fed individually once per day based on their daily energy requirements and water was available *ad libitum*.

### Parasite infection and sample collection

*Toxoplasma gondii* PRU strain belongs to the predominant genotype II reported in cats [16–18]. The capacity of the PRU strain to produce cysts in the brain of mice and oocysts in the gut of cats makes it a suitable candidate for experimental infections of cats [19]. *Toxoplasma gondii* PRU strain was maintained by passage through Kunming mice [20]. We used a low passage PRU strain to preserve the biological attributes and fidelity to the original strain. Brain cysts of *T. gondii* were examined microscopically and their number was adjusted to 100 cysts/ml in phosphate buffered saline (PBS, pH 7.4). Cats (*n* = 21) were randomly allocated to 7 groups (i.e. 3 cats per group). Six groups were subjected to infection, where each cat was infected by intragastric inoculation with 100 cysts in 1 ml sterile PBS. One group of cats (remained uninfected as a control group) received 1 ml of sterile PBS only. Cat intestinal tissue was collected at different time points [6, 12, 18, 24, 72 and 96 hours post-infection (hpi)] from cats infected with *T. gondii*. Intestinal tissue was also collected from three cats in the uninfected group. The collected tissues were rinsed trice in PBS and kept frozen in liquid nitrogen until further processing.

### RNA isolation and quantification

Total RNA from each intestine was extracted by TRIzol Reagent (Invitrogen Co. Ltd, San Diego, USA). RNA degradation and contamination were examined by electrophoresis on 1% agarose gels. RNA purity was checked by a NanoPhotometer® spectrophotometer (Implen, Westlake Village, CA, USA). RNA concentration was measured using a Qubit® RNA Assay Kit in a Qubit® 2.0 Fluorometer (Life Technologies, Carlsbad, CA, USA). RNA integrity was assessed using an RNA Nano 6000 Assay Kit and an Agilent Bioanalyzer 2100 system (Agilent Technologies, Santa Clara, CA, USA), demonstrating an RNA integrity number > 8.

### Confirmation of *T. gondii* infection in the intestines

RNA (1 μg) collected at each time point stated above were reverse-transcribed to a single strand cDNA using a PrimeScript™ RT reagent kit with gDNA Eraster (Takara, Dalian, China). Infection was detected in the intestinal tissues using quantitative real-time PCR (qRT-PCR), which targets surface antigen one (SAG1) of *T. gondii* using forward primer: 5'-CAC AGA GCC TCC CAC TCT TG-3' and reverse primer: 5'-AGA CTA GCA GAA TCC CCC GT-3'. qRT-PCR was performed using a 7500 system (ABI), employing SYBR® Premix *Ex Taq*™ II (Takara). The reaction was performed in a final volume of 20 μl containing 10.4 μl Premix *Ex Taq* II, 0.8 μl of each primer, 2 μl of cDNA template, and 6 μl of double-distilled water (ddH_2_O). PCR conditions were as follows: initial denaturation step at 95 °C for 30 s, followed by 40 cycles of 95 °C for 5 s and 60 °C for 34 s. All templates were examined in triplicate and controls without template were also included. The results of qRT-PCR were calculated by determination of the 2^−ΔΔ*CT*^ (where *CT* is the threshold cycle) (relative expression) level.

### Library preparation for sequencing

Three micrograms of RNA per intestinal sample were used as an input for the RNA sample preparations. Sequencing libraries were generated using an NEBNext® Ultra™ RNA Library Prep Kit for Illumina® (NEB, Ipswich, USA) following manufacturer’s recommendations and index codes were added to correlate sequences to their respective samples. The mRNA was purified from total RNA using poly-T oligo-attached magnetic beads. Fragmentation was performed using divalent cations under elevated temperature in NEBNext First Strand Synthesis Reaction Buffer (5×). First strand cDNA was synthesized using random hexamer primer and M-MuLV Reverse Transcriptase (RNase H). Second strand cDNA synthesis was subsequently performed using DNA Polymerase I and RNase H. Remaining overhangs were converted into blunt ends *via* exonuclease/polymerase activities. After adenylation of the 3' ends of DNA fragments, NEBNext Adaptors with a hairpin loop structure were ligated to prepare for hybridization. In order to select cDNA fragments preferentially ~150–200 bp in length, the library fragments were purified with AMPure XP system (Beckman Coulter, Beverly, USA). Then, 3 μl of USER Enzyme (NEB) were used with size-selected, adaptor-ligated cDNA at 37 °C for 15 min followed by 5 min at 95 °C before PCR. The PCR was performed with Phusion High-Fidelity DNA polymerase, Universal PCR primers and Index (X) Primer. PCR products were purified (AMPure XP system) and the quality of the libraries was assessed using an Agilent Bioanalyzer 2100 system. The clustering of the index-coded samples was performed on a cBot Cluster Generation System using TruSeq PE Cluster Kit v3-cBot-HS (Illumina, San Diego, USA) according to the manufacturer’s instructions.

### Differential expression analysis

Raw reads of fastq format were processed using in-house Perl scripts. Clean reads were obtained by removing reads adapters, poly-N containing reads and low-quality reads from raw data. The Q20, Q30 and GC content of the clean data were determined. All downstream analyses were based on the clean data. The *Felis catus* genome was used as the reference genome and gene model annotation files were downloaded from the cat genome website (ftp://ftp.ensembl.org/pub/release-76/fasta/felis_catus/dna/). Index of the reference genome was built using Bowtie v.2.2.3 and paired-end clean reads were aligned to *F. catus* reference genome using TopHat v.2.0.12. TopHat was selected as the mapping tool since it can produce a database of splice junctions based on the gene model annotation file and provides a better mapping result than other mapping tools [21]. HTSeq v.0.6.1 was used to count the read numbers mapped to each gene. Fragments per kilobase of transcript sequence per million base pairs sequenced (FPKM) of each gene was calculated in order to determine the level of gene expression. Differential expression analysis of two groups (three replicates per group) was performed using the DESeq R package (1.18.0) [22]. The *P*-values were adjusted using the Benjamini-Hochberg correction for multiple testing. Genes were determined to be significantly differentially expressed if they had a |log2(fold change)| > 0.58 and false-discovery rate (FDR) < 0.05.

Pearson’s correlation analysis of samples used for RNA sequencing was carried out to examine the correlation between gene expression levels among samples. The square of the Pearson’s correlation coefficient (*R*^2^) < 0.92 indicates optimal sampling selection and experimental conditions. The distribution of differentially expressed genes (DEGs) on the chromosomes of cat genomes was examined. Clustering analysis of the DEGs was performed to determine the co-expression pattern of genes at different time points after infection. The genes with the same or similar expression pattern were grouped together into a cluster. *K*-means cluster was achieved based on the relative expression level of DEGs log_2_(fold change).

### Gene Ontology (GO) and Kyoto Encyclopedia of Genes and Genomes (KEGG) analysis

GO enrichment analysis of DEGs was carried out using the *GOseq* R package [23]. All DEGs were mapped to GO terms in the database (http://www.geneontology.org/), and then gene numbers were calculated for every GO term using the hypergeometric test in order to obtain the significantly enriched GO terms for the DEGs; these were compared to the genomic background. GO terms with a corrected *P*-value less than 0.05 were considered significantly enriched by DEGs. KOBAS software was used to perform pathway analysis and to test the statistical enrichment of the DEGs in KEGG (http://www.genome.jp/kegg/) [24, 25]. This analysis was used to identify significantly enriched genes involved in metabolic or signalling pathways.

### Validation of the RNA-Seq data

The expression of 10 to 14 selected genes was investigated by qRT-PCR to confirm the RNA sequencing based transcriptional response of cat intestine to *T. gondii* infection. These genes were identified by sequencing analysis as differentially upregulated or downregulated following the infection. Total RNA was isolated from the intestinal tissues obtained from *T. gondii*-infected and uninfected (control) cats at different time points after infection using RNeasy Mini Kit (Qiagen, Hilden, Germany). DNase-digested total RNA (1 μg) was reverse-transcribed to single strand cDNA using the Primer Script™ RT Reagent Kit with gDNA Eraser (Takara). SYBR Premix *Ex Taq*™ II (Takara) was used to perform qRT-PCR on an ABI real-time PCR cycler (ABI 7500). Forward (F) and reverse (R) primers used to amplify the selected genes are listed in Table 1. The amplification reactions were performed using the following conditions: 95 °C for 10 min followed by 40 cycles of 95 °C for 15 s and 60 °C for 1 min. Melting curve analysis was performed using the following conditions: 1 min at 95 °C, 65 °C for 2 min and progressive increase from 65 to 95 °C to ensure that a single product was amplified in each reaction. The relative fold change in gene expression was calculated following actin normalization using the 2^−ΔΔCT^ method. The mean values of the control cats were used to calculate the fold change for the infected cats.

**Table 1:** Primers used in the qRT-PCR in the present study

Gene	Sequence (5'-3')	Primer length (mer)	Tm (°C)	GC %	Product length (bp)
β-actin	ATTCCACGGCACAGTCAAGG	20	63.39	55	110
	CACCAGCATCACCCCATTT	19	61.75	52.63	
RSAD2	CACCAGCGTCAACTACCACT	20	59.97	55	140
	AATATTCACCGGCTTCCTGC	20	58.32	50	
SYCN	AAGACGCGCAAGTTCTCGAC	20	61.28	55	117
	AAGGCATCAGTAACACCTGCAA	22	60.49	45.45	
CELA1	ATGCTACGCTTCTTGGTGCT	20	60.04	50	239
	CGGAAGGTCATTTTGCGGTC	20	59.83	50	
GKN2	CTTGAAGGTGGTATGCCTGGT	21	60	52.38	214
	CCTGGATGCGATGTAGCGAT	20	60.04	55	
TFF2	ATGCGTCATGGAAGTCTCGG	22	62.14	50	198
	TGACAGTCGTCAACGGACATC	24	61.04	45.83	
LYPF	TTACACCCGACAAACCCTGA	20	59.24	52.38	132
	CAGGTCTCCGGCCTTTATTCT	21	60.67	55	
CYP1A1	TCGATACCTACCCAACCCTG	20	58.22	55	246
	ACTGTGTCAAATCCAGCTCCG	21	60.61	52.38	
CLPS	TGCTCTGCCAAGACACTCTAT	21	58.82	47.62	222
	5'CAATCAGACAAGGGAGGTGCT	21	60	52.38	
CPA2	TGCTCAGTCATCTACCAAGCC	21	59.79	52.38	100
	ATCGACCTGTGTCCCTCAGT	20	60.25	55	
PRSS2	TACTTCTGCCGCCATGACTC	20	59.82	55	227
	TCACTTGGATGCGAGACTTGT	21	59.66	47.62	
CPA1	AACGATTTACCAAGCCAGCGG	21	61.55	52.38	210
	GCCGAAGGACCAGTTCAGTA	20	59.39	55	
FLNC	AGTGGTGCCACCCTGTAACC	20	62.06	60	181
	CTCTCGCACTTTCACTGGCT	20	60.32	55	
FN1	GAACACTAATGTCAACTGCCCA	22	58.85	45.45	125
	TGGACCTTGGCAGAGAATCC	20	59.38	55	
DES	ATGTCCAAGCCAGACCTCAC	20	59.67	55	240
	GGAATCGTTGGTGCCCTTGA	20	60.61	55	
ACTG2	GTATACTCGGTGCTCAAGCC	20	58.15	55	243
	TAGGATCCCTCGCTTGCTCT	20	60.11	55	
FABP6	CCCACAGCTACCAACGCTAC	20	60.74	60	185
	ATTCTGCTGGAGAGCATCTTCAAT	24	60.39	41.67	
MYH11	GCATGCTGCAAGATCGAGAG	20	59.42	55	147
	CTTGCGTGATGCTTGTGTCC	20	60.11	55	
FGB	CCCAATCAACCTTCGTGTGC	20	59.76	55	156
	TCCTCACATTCTTTGCCAGACA	22	59.89	45.45	
TAGLN	AACGGCGTGATTCTGAGCAA	20	60.23	47.2	158
	CCACCTGCTCCATTTGCTTG	21	59.78	50	
ALPI	ACACACCTCATGGGCCTCTT	20	61.14	55	199
	TCAGTGCCAGATAAGCCCTG	20	59.46	55	
CNN1	TGGCATCATTCTTTGCGAGTTC	22	59.84	45.45	153
	TCGAAAATGTCGTGGGGCTT	20	60.25	50	
LYPD2	TGAGATGCTACACCTGTCACG	21	59.80	52.38	123
	AGAAGGGGTACACTATCTCCAAG	23	58.71	47.83	
pCREB	CCACAACCATCCGTCCTTCT	20	62.26	55	137
	TGTGTGTGTGTGTGTCGGTATGT	23	62.26	47.83	
MMP	GACGACGATGAGCTGTGGA	19	61.02	57.89	115
	TGGTGTATTCCTTGCCGTTG	20	61.85	50	
FABP1	CTGATGAAGGTGCCAAGAACAA	22	62.46	45.45	132
	GCATTTCCTCACTATTTCCCACTC	24	62.3	45.83	

*Abbreviation*: Tm, melting temperature

## Results

### Confirmation of *T. gondii* infection

Detection and amplification of *T. gondii SAG1* gene was confirmed in all infected cats by qRT-PCR assay. The three cats in the control group were qRT-PCR-negative. The results also revealed that *SAG1* gene was amplified in intestinal samples of cats starting from 6 hpi and peaking at 96 hpi.

### RNA-Seq data

**Fig. 1:**
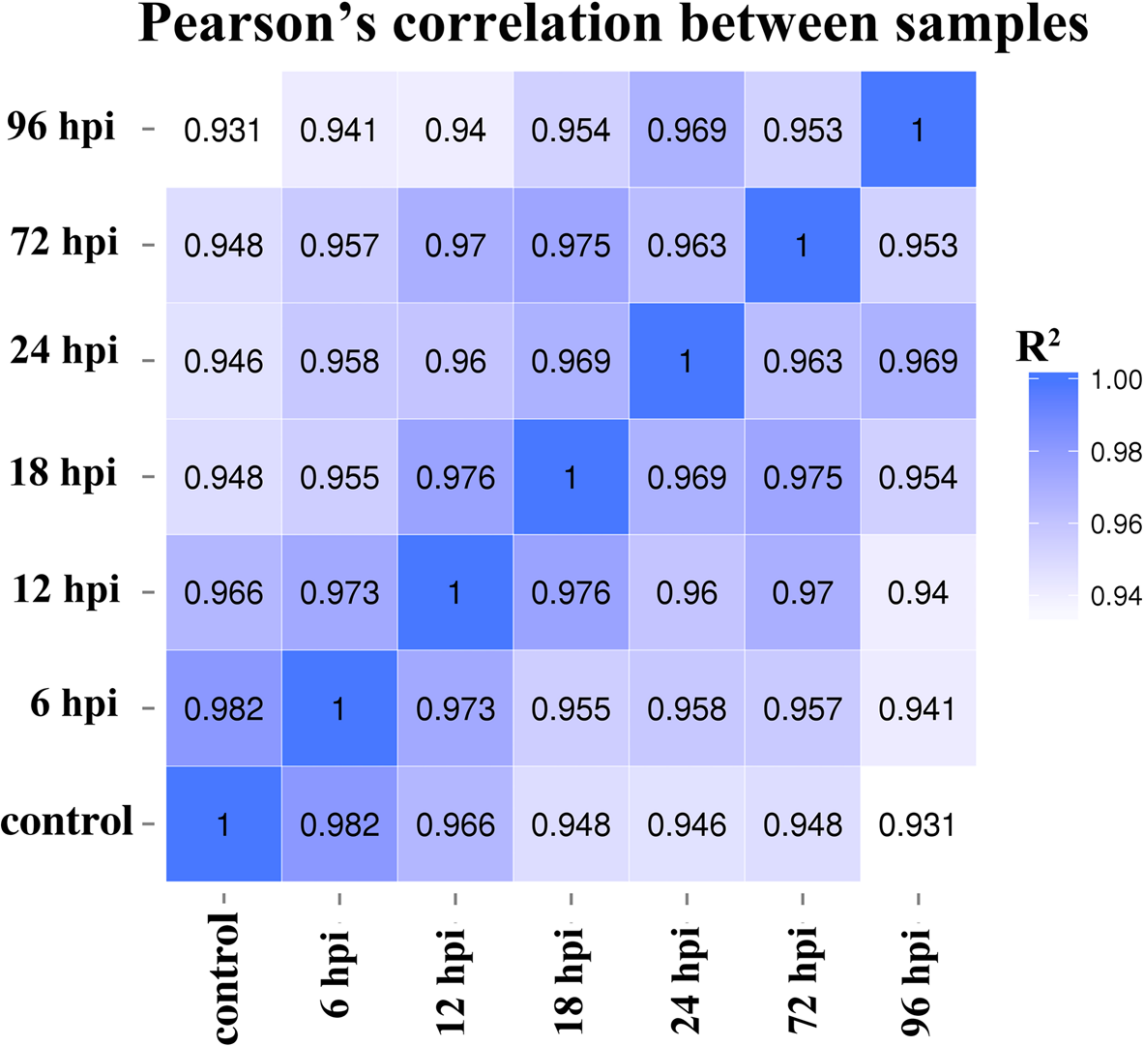
A heatmap showing the magnitude of the Pearson’s correlation coefficient matrix among different groups. The map shows that all correlations in this matrix are positive and of moderate to large value. Variables: 0 (control), 6, 12, 18, 24, 72 and 96 hours post-infection

**Fig. 2:**
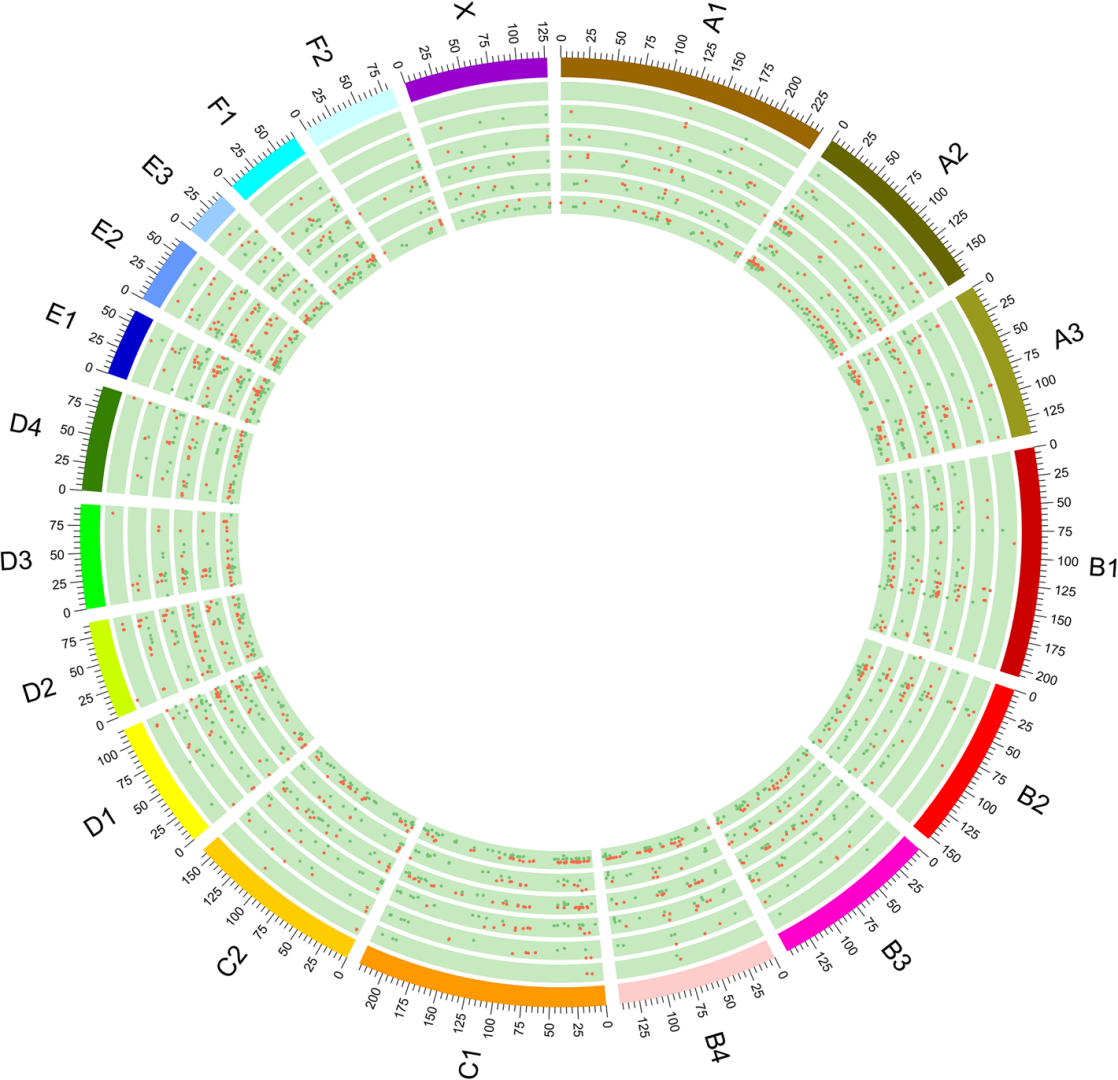
Chromosomal position of the differentially expressed genes (DEGs) implicated in *T. gondii* interaction with cat intestine. Circos plot shows the relative expression levels of DEGs in the cat chromosomes. Up- and downregulated genes were found on all cat chromosomes; however, the DEGs encoded by chromosome X were mainly downregulated. Chromosome number and bands are identified in the outermost ring. Other tracks from outer to inner compares the cat intestinal transcriptional response to *T. gondii* infection at 6, 12, 18, 24, 72, and 96 hours post-infection. Red and green color represents the upregulated and downregulated genes, respectively

We employed RNA-Seq to investigate the temporal gene expression patterns of cat intestine during early infection with *T. gondii* PRU strain. Over 25,000,000 raw reads were obtained from each intestinal sample and more than 24,000,000 clean reads were obtained after removing adaptors and low-quality reads. About 83% of the clean reads were mapped to the reference genome and more than 70% of the clean reads were located in the exon regions; the rest were in the introns or intergenic. Pearson’s correlation coefficient of gene expression among different time points was close to 1, indicating the high similarity of the gene expression patterns between samples (Fig. 1). The distribution of DEGs on cat chromosomes is shown in Fig. 2. The chromosomal location of DEGs across the different time points after infection showed more upregulated gene expression on chromosomes D2, D3 and E2, and a skewed pattern of downregulated genes on the X chromosome (Additional file 1: Table S1).

### Gene expression analysis

**Fig. 3:**
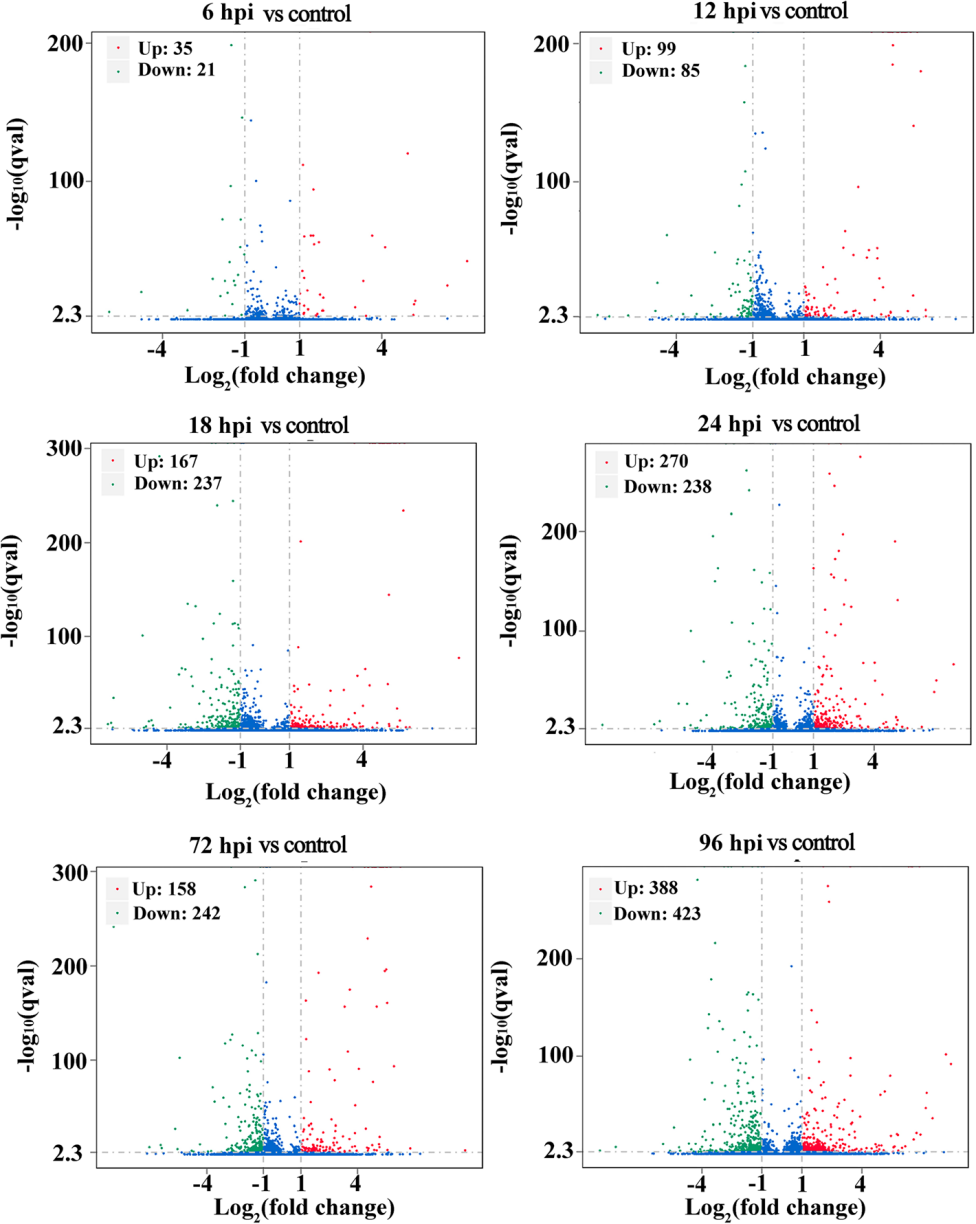
Volcano plots showing the transcriptional response of cat intestine to infection with *T. gondii* PRU strain at each of the indicated time points after infection compared to those of the uninfected controls. Differentially expressed genes (DEGs) are shown as red (upregulated) or green (downregulated) dots. Non-significant difference between the expressions of genes is indicated by blue dots. The X-axis represents the value of log_2_(fold change) and the Y-axis shows the value of -log_10_(qval)

**Fig. 4:**
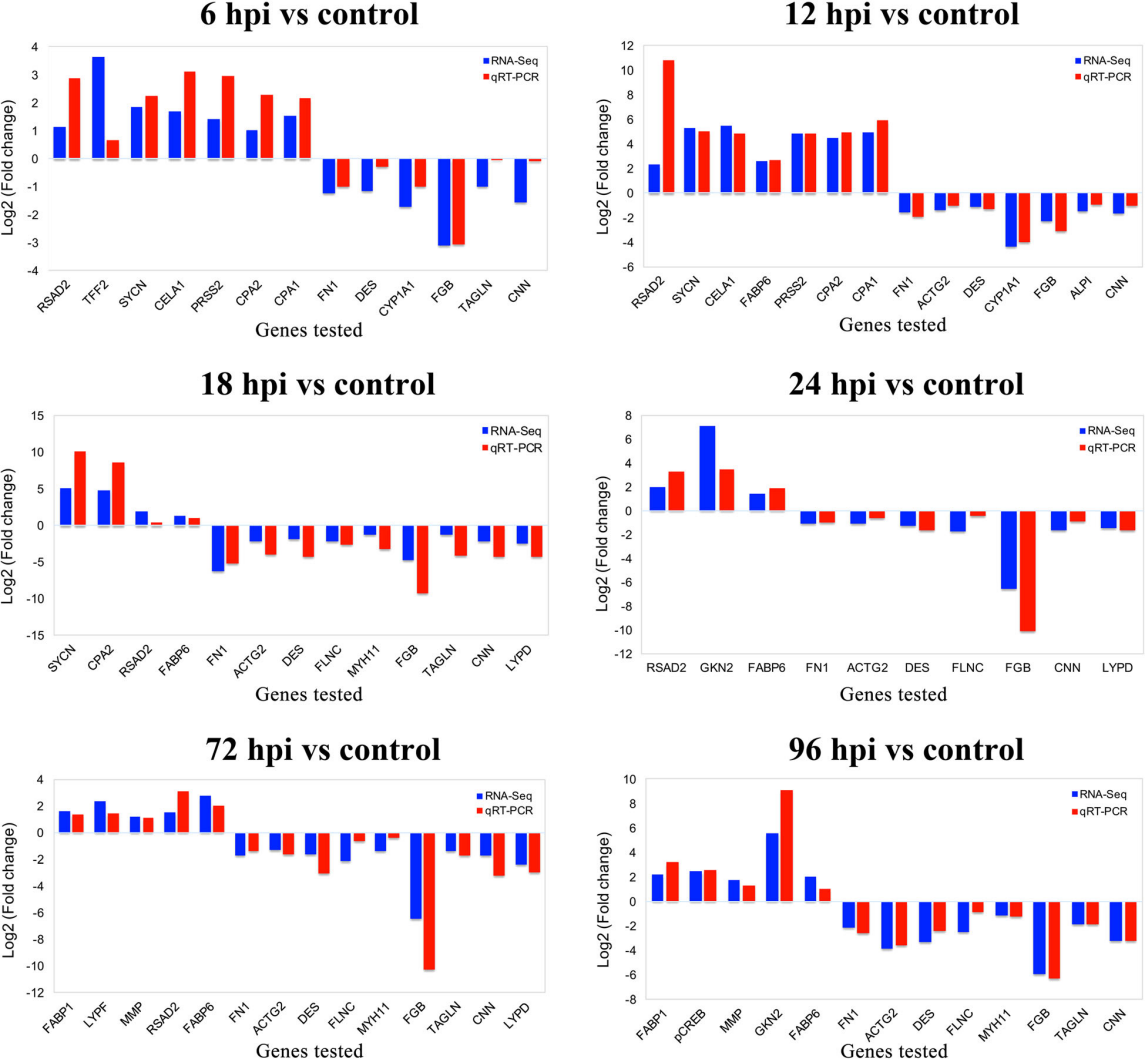
qRT-PCR-based verification of the gene expression obtained by RNA sequencing. Data of RNA-Seq were confirmed by qRT-PCR at 6, 12, 18, 24, 72 and 96 hours post-infection. The X-axis shows the name of genes tested and the Y-axis represents the relative expression for the genes examined

Differential gene expression analysis was performed by comparing the gene transcriptional level in response to *T. gondii* infection to that in the uninfected control samples at each time point after infection (FDR < 0.05 and |log2(fold change)| > 0.58). Transcriptome analysis revealed 2363 infection-specific DEGs, of which 56, 184, 404, 508, 400 and 811 genes were differentially expressed at 6, 12, 18, 24, 72 and 96 hpi, respectively (Fig. 3, Additional file 2: Table S2). qRT-PCR results of the examined set of genes at 6, 12, 18, 24, 72 and 96 hpi in cat intestine were consistent with the RNA-Seq results, confirming the validity of sequencing data (Fig. 4).

### Cluster analysis

**Fig. 5:**
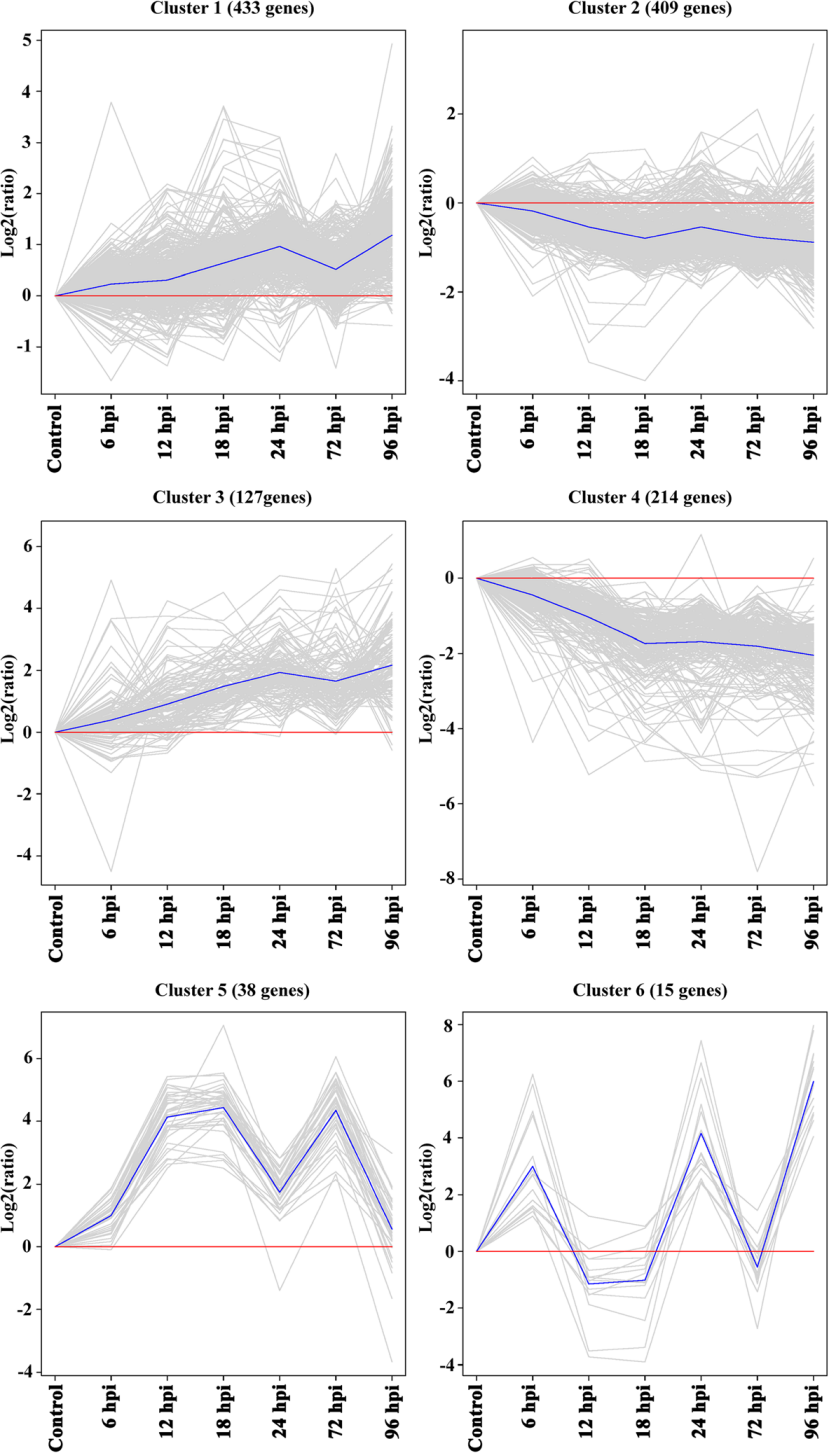
Expression pattern clustering from RNA-Seq analysis of 2363 DEGs in the intestine of cats infected with *T. gondii*. Differentially expressed, co-regulated genes from adjacent stage pairwise comparisons were analyzed using k-means clustering. The identified DEGs were grouped into 6 clusters based on the similarity of their expression. The six clusters are shown in a graphical format based on the pattern of expression at different time points after infection. The X-axis represents time points after infection and the Y-axis indicates the relative gene expression

**Fig. 6:**
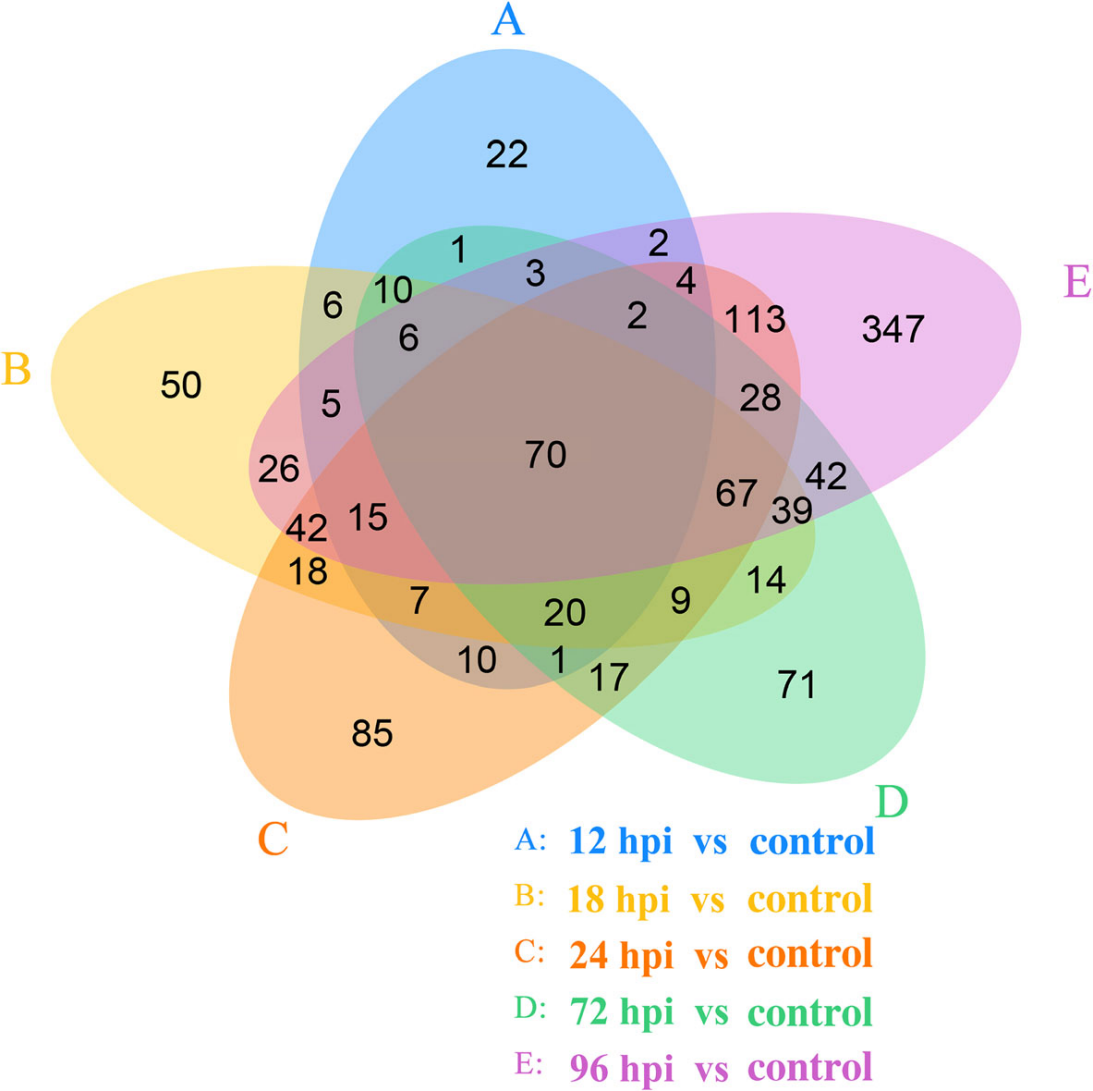
Venn diagram depicting the respective unique and shared differentially expressed genes among five *Toxoplasma gondii*-infected groups at 12, 18, 24, 72 and 96 hours post-infection. The result of 6 hpi was not shown because this two-dimensional diagram could not display more than five comparisons

Hierarchical cluster analysis using k-means grouped all DEGs into six clusters according to their expression pattern across the six time points post-infection (Fig. 5). Cluster 1 (containing 433 genes) and cluster 3 (127 genes) showed a progressive increase in the expression with time after infection. Genes in cluster 2 (409 genes) and cluster 4 (214 genes) were downregulated as *T. gondii* infection proceeds. Cluster 5 (38 genes) and cluster 6 (15 genes) showed a bi-phasic upregulated expressional pattern, but at different time points after infection. Venn diagram analysis revealed 70 genes that were differentially co-expressed in infected samples at all time points (12, 18, 24, 72 and 96 hpi) compared with control samples (Fig. 6). The highest number of specific DEGs was detected at 96 hpi (347), which was significantly higher than that of any other time point. The number of DEGs from 72 and 96 hpi were less than that from 24 and 96 hpi. The mechanisms that underpin these temporal changes of gene expressions remain to be investigated. These aforementioned results indicate that most of the upregulated genes were grouped in clusters 1, 3 and 5, whereas clusters 2 and 4 contained most of the downregulated genes.

### Gene ontology (GO) classifications

**Fig. 7:**
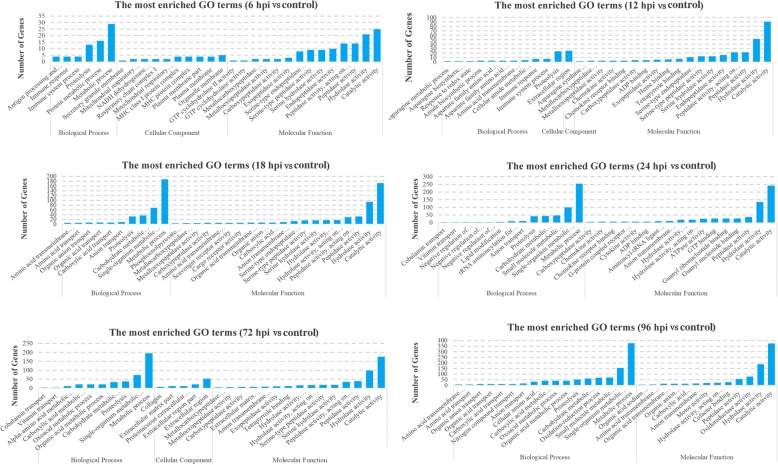
The gene ontology (GO) analysis of the DEGs. The 30 most significantly enriched GO terms for each time point after infected *versus* control are shown. The GO distributions are summarized in three main categories: biological process, cellular component and molecular function. The X-axis shows different GO terms and the Y-axis indicates the corresponding number of genes in each GO term

GO analysis showed that DEGs were overrepresented by GO terms involved in immune responses, such as chemokine receptor binding and cytokine activity. The top 30 differentially expressed GO terms of the DEGs in cat intestine are shown in Table 2. GO analysis of DEGs at various time points post-infection revealed the dynamic changes in biological processes, molecular function and cellular component during early stages of *T. gondii* infection in the cat intestine. The top 30 most enriched GO terms with the corresponding number of genes at each time point after infection are shown (Fig. 7). Catalytic activity and metabolic process were the most significantly enriched GO terms in molecular function category and biological process category, respectively. This finding was consistently observed in all of the six groups (6, 12, 18, 24, 72, and 96 hpi) compared to the uninfected control group, suggesting that these processes play fundamental roles in feline response to infection with this parasite.

**Table 2:** The top 30 differentially expressed GO terms between *T. gondii*-infected and uninfected cats

GO category	GO name	Gene number						FDR corrected *P*-value
		Cluster 1	Cluster 2	Cluster 3	Cluster 4	Cluster 5	Cluster 6	
Biological process	Carbohydrate_metabolic_process	22	28	4	27	1	0	2.03E-04
	Cellular amino acid metabolic process	20	8	8	6	0	0	0.014098
	tRNA metabolic process	20	5	5	3	0	0	0.04415
Cellular component	Extracellular region	30	27	38	6	11	2	3.84E-04
	Extracellular matrix	7	7	14	0	3	0	0.018194
	Extracellular matrix component	1	4	10	0	1	0	0.014935
	Myosin complex	6	2	8	0	1	0	0.021049
	Extracellular region part	13	11	19	0	5	0	0.022411
Molecular function	Catalytic activity	194	93	182	19	40	5	4.44E-16
	Hydrolase activity	95	52	93	15	13	3	3.02E-10
	Ion binding	137	49	117	6	23	1	0.044804
	Nucleotide binding	94	16	48	2	11	0	0.008417
	Small molecule binding	99	19	55	3	12	0	0.001499
	Anion binding	92	18	49	1	10	1	0.020323
	Carbohydrate derivative binding	83	16	42	3	10	0	0.026838
	Pyrophosphatase activity	52	6	24	0	4	0	0.030815
	Nucleoside-triphosphatase activity	51	6	23	0	4	0	0.043152
	Oxidoreductase activity	47	14	41	3	10	1	2.03E-04
	Cofactor binding	23	9	23	2	8	0	0.001491
	Cargo receptor activity	4	3	4	0	0	0	0.001611
	Chemokine receptor binding	5	0	1	0	4	0	0.002826
	Coenzyme binding	15	4	12	1	3	0	0.008153
	G-protein coupled receptor binding	5	0	1	0	4	0	0.012537
	Motor activity	12	2	9	0	1	0	0.012498
	Metalloexopeptidase activity	0	2	2	3	0	0	0.013723
	Metallocarboxypeptidase activity	0	2	1	3	0	0	0.036387
	Tetrapyrrole binding	4	4	7	1	4	0	0.029038
	Cytokine activity	5	0	1	0	4	0	0.033712
	Peroxiredoxin activity	4	0	1	0	0	0	0.037208
	Adenylylsulfate kinase activity	2	1	2	0	0	0	0.044538

### Functional analysis of the transcriptional response of cat intestine to *T. gondii* infection

**Fig. 8:**
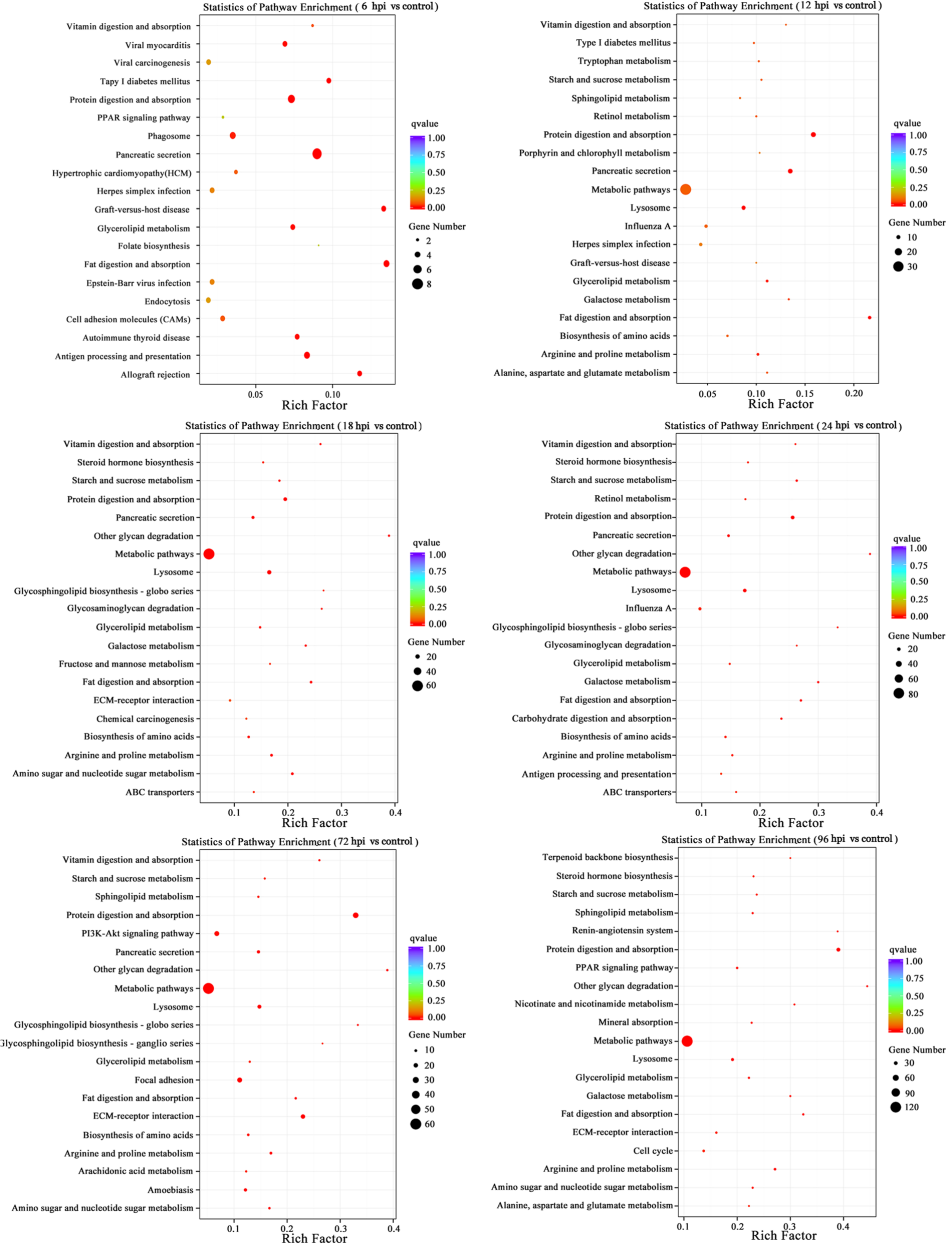
Scatter plots of the enriched KEGG pathways statistics. The *q*-value is the corrected *P*-value (range from 0 to 1). The colour and size of the dots represent the range of the *q*-value (level of significance) and the number of DEGs mapped to the indicated pathways, respectively. The X-axis shows the enrichment factor; the Y-axis corresponds to the KEGG Pathway. The top 20 enriched KEGG pathways of the indicated groups are shown. Rich factor is the ratio of the DEG number to the total gene number in a certain pathway

Compared to controls, 145 genes were assigned to 62 pathways at 6 hpi, 386 genes were involved in 141 pathways at 12 hpi, 674 genes were involved in 201 pathways at 18 hpi, 890 genes were involved in 206 pathways at 24 hpi, 673 genes were involved in 189 pathways at 72 hpi, and 1321 genes were involved in 238 pathways at 96 hpi. The top 20 most overrepresented pathways in each group are shown in Fig. 8. Interestingly, pathways related to metabolism were enriched in samples from all time points except at 6 hpi. These results agree with the results of GO analysis of DEGs and show that fewer host pathways were identified within 6 h of infection to be uniquely affected by *T. gondii* infection. As shown in Table 3, 36 KEGG pathways were differentially expressed in cat intestine following *T. gondii* infection.

**Table 3:** Total differentially expressed KEGG pathways overrepresented/enriched by the differentially expressed genes in cat intestine in response to infection with *T. gondii*

KEGG pathway	Gene number						FDR corrected *P*-value
	Cluster 1	Cluster 2	Cluster 3	Cluster 4	Cluster 5	Cluster 6	
Protein digestion and absorption	1	16	1	10	7	1	2.66E-15
Lysosome	3	9	1	13	0	1	4.44E-06
Mineral absorption	0	10	1	3	0	0	5.75E-05
ECM-receptor interaction	0	13	1	5	0	0	0.000222
Pancreatic secretion	4	6	0	0	9	0	0.000232
Galactose metabolism	2	2	1	6	0	0	0.000677
Other glycan degradation	0	3	0	5	0	0	0.001051
Amino sugar and nucleotide sugar metabolism	2	4	1	6	0	0	0.001526
Arginine biosynthesis	1	2	1	4	0	0	0.002527
Bile secretion	1	8	2	3	0	0	0.00304
Alanine, aspartate and glutamate metabolism	1	4	2	3	0	0	0.003204
Retinol metabolism	2	4	0	4	0	0	0.005355
Focal adhesion	4	14	1	7	0	0	0.010981
Starch and sucrose metabolism	0	2	1	5	1	0	0.011597
Sphingolipid metabolism	0	4	1	5	0	0	0.013042
Cell cycle	17	0	1	0	0	0	0.014158
Carbohydrate digestion and absorption	0	2	1	5	1	0	0.014633
Proximal tubule bicarbonate reclamation	1	5	1	0	0	0	0.014947
Arginine and proline metabolism	1	5	0	4	0	0	0.01764
Glycosaminoglycan degradation	1	1	0	4	0	0	0.01764
ABC transporters	2	5	0	2	0	0	0.021963
Glycosphingolipid biosynthesis - globo series	0	1	0	4	0	0	0.023611
Steroid hormone biosynthesis	1	2	1	3	0	1	0.024807
Fructose and mannose metabolism	1	4	1	2	0	0	0.024807
Glycolysis / Gluconeogenesis	2	5	1	3	0	0	0.024807
Chemical carcinogenesis	1	6	0	2	0	0	0.024807
Glutathione metabolism	3	5	1	0	0	0	0.024807
Steroid biosynthesis	6	0	0	0	0	0	0.025842
Drug metabolism - cytochrome P450	1	6	0	1	0	0	0.025842
Complement and coagulation cascades	6	2	3	1	0	0	0.026353
p53 signaling pathway	6	2	3	0	0	0	0.026976
Glycine, serine and threonine metabolism	4	1	2	1	0	0	0.027354
Renin-angiotensin system	1	3	0	2	0	0	0.027354
Pyrimidine metabolism	10	1	2	0	0	0	0.03345
Amoebiasis	3	7	0	4	0	0	0.034505
Nitrogen metabolism	3	0	1	1	0	0	0.034505

## Discussion

This study aimed to investigate how gene expression in the cat intestine is altered during early infection with *T. gondii*. We used RNA sequencing analysis to determine any temporal changes in the transcriptional response of the intestine of cats infected with *T. gondii* at 6, 12, 18, 24, 72 and 96 hpi. Our results showed that 2363 genes were differentially expressed in the intestine of infected cats compared to uninfected control cats.

GO enrichment and KEGG pathway analyses showed that DEGs involved in the metabolic process and catalytic activity were the most enriched at all time points post-infection. A previous study has reported the upregulation of cell growth/maintenance (Translation and Transcription GO categories) and metabolism (Glycolysis and TCA Cycle GO categories)-related genes in the *T. gondii* merozoite stage during intestinal infection of mice [26]. This intraepithelial intestinal stage of *T. gondii* proliferates asexually using endopolygeny, whereby multiple daughters are generated from a single parental organism [27]. The results of the present and previous studies underscore the high bioenergetic demands of the growing parasite, in order to adapt to the low oxygen tension in the cat’s intestine, and sustain its proliferation [28].

Our results also showed the downregulation of genes involved in the class I major histocompatibility complex (MHC I) pathway, at 6 and 12 hpi. MHC I is composed of the polymorphic heavy chain, β2-microglobulin, and antigenic peptides. The latter can be derived from endogenous proteins and from proteins of this intracellular parasite. These proteins are degraded by the proteolytic proteasome complex, then transported into the endoplasmic reticulum (ER) where they associate with the class I molecule to form MHC I. One of the physiological functions of MHC I is recognition by the peptide-specific T-cell receptors of cytolytic T lymphocytes (CTLs). These cells are known to play critical roles in controlling *T. gondii* infection, by limiting the reactivation of latent infection and parasite proliferation during acute infection [29–31]. CTLs eliminate infected cells by recognition of foreign antigens that are processed in a proteasome-dependent pathway and presented by MHC I [32]. Therefore, the downregulation of MHC I genes during the first 12 hours after infection can reduce the antigen-specific CTL cell-mediated killing of *T. gondii*-infected cells [33]. Interferon gamma (IFN-γ) plays a critical role in innate immunity during acute *T. gondii* infection [34]. PA28, a proteasome activator induced by IFN-γ, has been implicated in MHC I antigen processing. The upregulation of the *PA28* gene, observed at 24 hpi, indicates that levels of this IFN-γ-inducible activator protein, PA28, had increased to enhance the cat’s defense response. PA28 promotes antigen processing and presentation, through recruiting more immunoproteasomes to proteasomes, and processing more parasite antigens during acute infection [35, 36]. Antigenic peptides resulting from proteasomal degradation are translocated into the ER lumen *via* transporters associated with antigen processing (*TAP1/2*) [37]. Therefore, the upregulation of the *TAP1/2* gene, observed at 24 hpi, may have also enhanced the cats’ intestinal immune response to *T. gondii* infection.

The molecular chaperones heat-shock proteins (HSPs) can stimulate cells of the innate immune response by serving as ‘danger’-signaling molecules [38]. After HSP70 binds to the surface of antigen presenting cells, MHC I presentation of the HSP-bound cytosolic antigen occurs, mediated by a transporter associated with antigen processing (TAP) [39]. We did not find any significant changes in the regulation of MHC I gene expression at 18 and 24 hpi; however, *hsp70* and *hsp90* genes were upregulated at both time points, in addition to *PA28* and *TAP1/2* genes at 24 hpi. HSP70 and HSP90 are peptide-binding proteins, and are associated with antigenic epitopes [40]. The upregulation of the HSP70/HSP90 complex may support the immune response, through enhancing antigen processing and presentation *via* proteasome and enhancing cell viability. *hsp70* is expressed in response to a variety of pathological stimuli, and allows the cell to survive lethal insults [41]. The interplay between HSP70 and HSP90 is of crucial importance for cell viability [42]. *Toxoplasma gondii* may benefit from the upregulation of *hsp70*, by maintaining the integrity of surrogate host cells, in order to complete its own growth, and to evade immunological detection. A connection between the upregulation of *HSP* genes and the division of schizont nucleoli has been suggested as nuclear division was not detected after 24 hpi in cat intestine [43].

These results support the hypothesis that *T. gondii*, through the downregulation of MHCI- related genes evades the immune response in order to facilitate its own growth in the intestine of cats during the first 12 hpi. Host cells, *via* increasing the expression of *HSP70/90*, *PA28*, *TAP*, and *TAP1/2* genes, deploy chaperones, immunoproteasomes and transporters to limit infection, through promoting antigen processing and presentation. In the present study, as infection progressed, the expression of MHC I genes was elevated at 72 and 96 hpi, indicating that the cats may have mounted a CTL response, mediated by MHC I, to limit replication of the parasite. Infecting cultured rat-intestinal epithelial cells with mature sporozoites, induced an elevated expression of genes associated with tumor necrosis factor alpha (TNFα) signaling, *via* NF-κB [44]. This transcriptomic change was not observed in our study, suggesting that anti-*T. gondii* intestinal immunity can vary between different hosts and different parasite stages, and based on whether the infection was established under *in vitro* or *in vivo* conditions.

Fatty acid binding proteins (FABPs) are released from the cytoplasm following the loss of enterocyte membrane integrity. *Toxoplasma gondii* can damage cell integrity, leading to the release of FABPs into the circulatory system. Ileal FABP (Il-FABP), which is located at the distal part of the small intestine, can mediate the uptake of fatty-acids. Peroxisome proliferator-activated receptors (PPARs) are activated by fatty acids and their derivatives. Il-FABP was upregulated in the intestine of cats from 12 to 96 hpi, but was downregulated at 6 hpi. This suggests a correlation between infection-induced alterations in the expression of FABP, and its potential lipid-metabolizing capacity [45] on the activation of PPARs, and the subsequent influence on key biological processes, such as the regulation of glucose, lipid homeostasis, cell survival, inflammation, proliferation and differentiation.

Through metabolic activation caused by phase I enzymes in conjugation with phase II enzymes, xenobiotic compounds such as drugs and chemical pollutants can be eliminated in the urine or bile by phase III transporters [46]. Phase I enzymes include the cytochrome P450 (CYP) superfamily, whereas phase II conjugating enzymes include some enzyme superfamilies; for example sulfotransferase, glutathione S-transferase (GST) and uridine diphosphate-glucuronosyltransferase (UGT). Some CYPs, for example CYP1A1, CYP1A2, CYP1B1 and CYP2A6, are procarcinogen-bioactivating enzymes. By metabolizing xenobiotics into reactive oxygenated intermediates (ROMs), which can cause genotoxicity and mutation by covalently binding to nucleic acids and proteins, CYPs can trigger tumor development [46]. In this study, the expression of the *CYP1A1* gene was downregulated from 6 to 96 hpi, except at 72 hpi, and the *GSTA2* gene was upregulated from 18 to 96 hpi. While downregulated *CYP1A1* can reduce the production of ROMs, thus minimizing DNA and protein damage, the upregulation of *GSTA2* may allow cytotoxic xenobiotics to accumulate, possibly triggering tumor development. This may be a mechanism *via* which *T. gondii* contributes to the development of some forms of cancer [47].

The expression of *UGT1A* was decreased from 12 to 96 hpi; however, no significant change in the gene expression was observed at 6 hpi. The UGT1A1 enzyme can detoxify many endogenous and exogenous compounds [48]. For example, irinotecan is widely used for the treatment of metastatic colorectal cancer and is metabolized by esterase to form a SN-38, which is further conjugated to UGT1A1. Patients with the UGT1A1 variant have poor metabolism of SN-38 and are thus prone to irinotecan toxicity because of the difficulty of SN-38 excretion from body in the non-toxic SN-38G form [49]. It is possible to hypothesize that cancer patients concurrently infected with *T. gondii*, if administered irinotecan, may experience serious toxicity due to limited detoxification and increased accumulation of SN-38 caused by reduced expression of *UGT1A*. The expression of *UGT1A* during the *T. gondii*-host interaction raises a new question about the potential impact of parasite infection on irinotecan-treated cancer patients.

A link between *T. gondii* infection and Alzheimer’s disease (AD) has also been previously suggested [50–52]. We were intrigued by the observation that the neutral endopeptidase (*NEP*), amyloid-β precursor protein (*APP*) and *APOE* genes were significantly downregulated at 24, 72 and 96 hpi, respectively. The altered expression of the *NEP*, *APP* and *APOE* genes may impact the metabolism of endogenous amyloid-β, putatively a main contributor to AD [53–55]. Here, the downregulation of *NEP*, *APP* and *APOE* genes at 24, 72 and 96 hpi suggests diverse means by which early *T. gondii* may contribute to the development of AD. In this study, we have not quantified the extent of *T. gondii* infection in each cat, nor considered the impact of parasite burden on the hosts’ response. Therefore, the differences in host transcript abundance, observed over the course of infection, might have been influenced by changes in parasite burden or changes in parasite life stage development. Quantification of parasite burden, either through qPCR or analyzing parasite reads in RNA-Seq data, should be used to address this issue in future investigations. In addition, looking at differences in host transcript abundance during parasite life stage transitions throughout the course of infection, may provide critical insights in to how the feline host supports sexual reproduction.

## Conclusions

Whole-transcriptome profiling, using RNA-Seq technology, of the cat’s intestine following infection with *T. gondii* improved our understanding of the signaling pathways that mediate the response of cat intestine to early infection. Comparing the infection groups to the PBS-treated control group, 56, 184, 404, 508, 400 and 811 significantly DEGs were identified at 6, 12, 18, 24, 72 and 96 hpi, respectively. Our data suggest that *T. gondii* can modulate immune response and metabolic pathway to facilitate its development and survival in cat intestine. At 6 and 12 hpi, downregulation of MHC I genes may contribute to the establishment of infection in cat intestine during this early stage of host-parasite interaction. To counter the infection, the cat leverages chaperones, immunoproteasomes and transporters, through the upregulation of *HSP70/90*, *PA28*, *TAP*, and *TAP1/2* genes, in order to promote potent antigen-specific immunity. Interestingly, *T. gondii* altered the expression of genes which may have relevance to other processes, such as xenobiotic metabolism and the pathogenesis of AD. Elucidation of the roles of these pathways in the protective immunity against *T. gondii* infection may reveal new targets for vaccine design and therapeutic interventions to break the parasite transmission cycle in the definitive host.

## Additional files

Additional file 1: **Table S1.** Temporal changes in the number of the differentially expressed genes by cat chromosomes. (XLSX 12 kb)

Additional file 2: **Table S2.** Differentially expressed genes at 6, 12, 18, 24, 72 and 96 hours post-infection (hpi) compared with the control, uninfected cats. (XLSX 259 kb)

## Abbreviations

AD: Alzheimer’s disease
AIDS: Acquired immune deficiency syndrome
APOE: Apolipoprotein E
APP: Amyloid precursor protein
CYPs: Cytochrome P450s
DC: Dendritic cells
DEGs: Differentially expressed genes
ER: Endoplasmic reticulum
FABPs: Fatty acid binding proteins
FDR: False discovery rate
GO: Gene ontology
GSTs: Glutathione S-transferases
hpi: Hours post-infection
HSP70: 70 kilodalton heat-shock proteins
HSP90: 90 kilodalton heat-shock proteins
IEL: Intraepithelial T lymphocytes
IFN-γ: Gamma interferon
IL 4/5/13/17: Interleukin 4/5/13/17
ILC: Innate lymphoid cells
KEGG: Kyoto Encyclopedia of Genes and Genomes
LP: Lamina propria
M cells: Microfold cells
MAT: Modified agglutination test
MHC I: Major histocompatibility complex I
NEP: Neutral endopeptidase
NK: Natural killer cells
PBS: Phosphate buffered saline
PPARs: Peroxisome proliferator-activated receptors
PSEN1: Presenilin-1 gene
PSEN2: Presenilin-2 gene
qRT-PCR: Quantitative reverse-transcribed polymerase chain reaction
RNA-Seq: RNA sequencing
ROMs: Reactive oxygenated intermediates
TAP: A transporter associated with antigen processing
TCR: T cell receptor
UGT1A1: Uridine diphosphate glucuronosyltransferase A1
